# Sexual size dimorphism in the evolutionary context of facultative paedomorphosis: insights from European newts

**DOI:** 10.1186/1471-2148-9-278

**Published:** 2009-12-02

**Authors:** Mathieu Denoël, Ana Ivanović, Georg Džukić, Miloš L Kalezić

**Affiliations:** 1 Laboratory of Fish and Amphibian Ethology, Behavioural Biology Unit, University of Liège, 22 Quai van Beneden, 4020 Liège, Belgium; 2 Institute of Zoology, Faculty of Biology, University of Belgrade, Studentski trg 16, 11000 Belgrade, Serbia; 3 Institute for Biological Research "Siniša Stanković", University of Belgrade, Bulevar despota Stefana 142, 11060 Belgrade, Serbia

## Abstract

**Background:**

Sexual size dimorphism (SSD) is a key evolutionary feature that has been studied in many organisms. In a wide range of species, this pattern is more complex because of polymorphism within each sex. However, it is not known whether the magnitude and direction of SSD could be affected by alternative developmental trajectories within sexes. Our aim was to test whether an intrasexual polymorphism, facultative paedomorphosis (a process in which the development of somatic and gonadal tissues differs in alternative morphs), could affect SSD variation patterns in European newts.

**Results:**

We report here the first evidence that SSD varies depending on the paedomorphic or metamorphic ontogenetic pathway. In species with a consistent female-biased SSD, paedomorphosis decreased the SSD level, but did not affect its direction. In species with moderate female-biased SSD or variable SSD patterns, paedomorphosis changed the magnitude, or both the magnitude and the direction, of SSD.

**Conclusion:**

Our study highlights the importance of developmental processes for shaping SSD patterns in populations in which contrasting life-history pathways evolved. European newts express different SSD patterns depending on their developmental pathway (i.e., metamorphosis versus paedomorphosis), as well as their species and population. These findings emphasize the importance of studying alternative morphotypes, which are found in a wide range of animal groups, to understand the evolution of SSD.

## Background

The difference between the sexes in terms of body size is a fundamental characteristic that can lead to important biological insights. Selective forces have driven the size of males and females in opposite directions as a result of competitive advantages in ecology (food and habitat use), fecundity, or sexual selection (size-related mate choice and intrasexual competition in combats) [1,2]. On the other hand, proximate determinants can be related to contrasting growth patterns in the two sexes before and after maturation [3]. This inherent link between developmental processes and the evolution of sexual size dimorphism (SSD) suggests that developmental polymorphisms could affect variation in SSD. Despite the diversity of polymorphisms in living species [see e.g. [4]], this issue remains a neglected area of research. The main studied example is the horn dimorphism in beetles, in which males and females, but also alternative male morphs differ in the size of horns [5]. When polymorphisms affect developmental dissociations between gonadic and somatic traits, these heterochronic processes could alter the magnitude of SSD, as sex differentiation can be also understood as a result of heterochronic changes [6-8]. In such cases, alternative phenotypes follow different ontogenetic pathways during which the exhibition of sex differences could be altered by changes in the rate, or timing of developmental events. Moreover, because heterochronic patterns are considered to be important possible steps towards speciation [9-11], understanding of the link between SSD and heterochrony is of primary importance.

Paedomorphosis in tailed amphibians implies the existence of two morphs that differ by the retention of gills at the adult stage, i.e., a mixture of larval and mature characters [12,13]. The ancestral character and the most widespread ontogenetic pathway is metamorphosis, in which aquatic gilled larvae metamorphose into juveniles that mature on land. Alternatively, paedomorphosis results in the sexual maturation of larvae without metamorphosis [14]. Such a discrete life-history polymorphism has both genetic and environmental components, and it is usually viewed as an adaptation to different environments in time and space [for the most recent review see [13]]. Because these morphs coexist within a single population (i.e., paedomorphosis is facultative rather than obligate, which would result in only one morph), this pattern offers the opportunity to study within-population variation of sexual dimorphism in body size and the effect of developmental variation on SSD.

European newts have served as important models for the study of SSD due in large part to their considerable variation in both extent and direction of intersexual body size differences [15-17]. Nevertheless, the association between sexual size dimorphism and facultative paedomorphosis has not yet been explicitly documented in these tailed amphibians. Among European newts, three species in particular are known to exhibit facultative paedomorphosis (all previously included in the genus *Triturus*): the smooth newt (*Lissotriton vulgaris*, Linnaeus, 1758), the palmate newt (*Lissotriton helveticus*, Razoumowski, 1789), and the alpine newt (*Mesotriton alpestris*, Laurenti, 1768). The high incidence of both morphs in particular populations of these three species [18,19] is a prerequisite for analysing the effects of alternative life cycle pathways on SSD at both the species and population levels. In addition, the three analysed species exhibit different patterns of SSD, which enables studying influences of facultative paedomorphosis on intersexual differences in body size within the framework of varied SSD patterns. The alpine newt has the most pronounced female-biased SSD (i.e., females larger than males), the palmate newt females are typically larger than males, and the smooth newt has variable SSD with a high degree of interpopulation variation in both the extent and direction of SSD [16,17].

In the present study, our objective was to investigate patterns of SSD variation by studying populations of the three European species in which a large number of paedomorphs and metamorphs can be found syntopically (paedomorphosis is too rare in other European newt species to use them in studying SSD). More specifically, we aimed at determining (1) whether there is an effect of paedomorphosis on sexual size differences; and (2) whether this pattern is species-specific and/or population-specific. Because facultative paedomorphosis implies the retention of larval traits in the adults, we expect a change in the magnitude of SSD between morphs. Particularly, we hypothesised that paedomorphs with underdeveloped bodies would exhibit less pronounced SSD patterns than metamorphs.

## Methods

### Sample and measures

The alpine newt and the smooth newt were examined from the Herpetological Collection of the Institute for Biological Research "Siniša Stanković", Belgrade, Serbia. All these individuals were preserved and stored in the collection before 1994. Palmate newts were sampled directly in the field in 2008. Three populations of each species were examined (for locality data and sample sizes, see Table 1). They were chosen in order to provide a large number of individuals of both sexes of both morphs. These populations came from the hotspots of paedomorphosis, notably the Montenegrin karst area [18] and Larzac in France [19].

**Table 1:** Locality data, sample sizes and body length (BL) for each morph and sex.

		Metamorphs				Paedomorphs			
		**Females**		**Males**		**Females**		**Males**	
**Species and population**	**Geographic coordinates**	***n***	**BL ± SE**	***n***	**BL ± SE**	***n***	**BL ± SE**	***n***	**BL ± SE**

**Alpine newt**									
Zminičko lake	442° 59' N, 19° 15' E	10	52.8 ± 2.0	19	44.3 ± 3.6	18	47 ± 3.8	9	42.3 ± 2.3
Bukumirsko lake	42° 36' N, 19° 33' E	14	50.8 ± 2.2	18	44.7 ± 2.2	49	50.7 ± 2.6	49	45.3 ± 2.2
Manito lake	42° 48' N, 19° 14' E	46	55.1 ± 2.4	49	46.9 ± 1.7	50	49.7 ± 3.0	36	44.8 ± 2.5
									
**Palmate newt**									
Sauvie	43°51' N, 3°35' E	15	44.7 ± 1.8	15	38.4 ± 1.6	15	39.2 ± 4.4	15	34.0 ± 3.6
Serre de la Labagne	43°57' N, 3°32' E	15	39.5 ± 2.1	15	35.5 ± 1.9	15	35.4 ± 2.4	15	34.7 ± 2.2
Azirou-croix	43°46' N, 3°29' E	15	40.1 ± 2.7	15	34.7 ± 1.0	15	38.6 ± 2.2	15	35.6 ± 2.6
									
**Smooth newt**									
Velika Osječenica	42° 58' N, 14° 40' E	50	43.2 ± 2.4	42	41.4 ± 2.1	46	39.9 ± 2.0	44	37.1 ± 1.7
Bag	44° 58' N, 14° 40' E	18	37.4 ± 2.5	10	37.9 ± 1.8	41	39.2 ± 1.7	39	37.4 ± 1.6
Hrastovača	46° 41' N, 18° 37' E	17	32.9 ± 3.2	23	31.8 ± 2.5	20	33.4 ± 2.6	35	34.2 ± 2.5

To investigate variability in body size and shape between the sexes and morphs, the following seven morphometric traits were scored: body length (BL, from the tip of the snout to the anterior margin of the cloaca), distance between legs (DL, from the posterior insertion of forelimbs to the anterior insertion of hind limbs), head width (HW), head length (HL), forelimb length (FLL), hind limb length (HLL) and tail length (TL). All measures of the alpine and smooth newts (paedomorphic and metamorphic individuals) were taken on alcohol-preserved specimens. The specimens of palmate newt were measured in a field laboratory on phenoxyethanol-anesthetised individuals (0.1%) and released in their study sites within one day of capture.

### Statistical analyses

In tailed amphibians, body size is most frequently assessed by snout-vent length, which is defined as the linear measure from the tip of the snout to the cloaca. However, this single linear measure might not fully describe size differences in overall body architecture [20]. To explore overall differences in body size between sexes, we included seven body measures in a principal component analysis (PCA), computing a linear summary of covariation in a set of linear size measurements and therefore providing an approximation of overall size [21]. The PCA was performed on a pooled dataset (sex and morphs of all three species) of log-transformed data for the seven morphological measures. The variation in PC1 scores between sexes, morphs and populations was explored with a factorial ANOVA within each species separately. We chose a multivariate sexual size dimorphism index (SSD I_PC1 _= mean female PC1score - mean male PC1score) as a measure of sexual dimorphism. Also, to estimate differences in size between morphs, we used mean "size" PC1 scores of paedomorphs and metamorphs of the same sex and calculated a heterochronic size dimorphism index (HSD I_PC1 _= mean metamorph PC1score - mean paedomorph PC1score) as a measure of size dimorphism among morphs of the same sex. Differences in the level of SSD between morphs were calculated as the difference between the calculated SSD indices (Paedomorphosis effect index, PE: SSD I_PC1 _metamorph - SSD I_PC1 _paedomorph).

A possible source of error may come from discrepancies in the sample sizes. We used a bootstrap procedure to generate 1000 samples. A "bootstrap sample" of *m *specimens (*m *= *n*, or *m *= 15 if *n *> 15) was repeatedly drawn randomly, with replacement, from the original sample (Pop Tools, version 2.7). PCA analyses were performed on each of 1000 generated datasets. We calculated the mean "size" PC1 scores for males and females in each population and morph. The SSD Index, HSD Index and PE Index were calculated for each of the bootstrapped datasets. To compare SSD between morphs, we used a bootstrap test against the null hypothesis that the morphs share the same SSD Index. To simulate this null hypothesis, the data for each pair of morphs were merged into a common pool of specimens. Bootstrap samples were then repeatedly drawn and SSD Index was computed. The magnitude of the difference in SSD was then compared to the value obtained from the original data. For each pair-wise combination, 1000 rounds of bootstrap re-sampling were used. If the difference between the means of pairs of bootstrap samples exceeded the observed differences in at least 5% of the total number of iterations, we could reject the null hypothesis that the means are equal [22].

All statistical analyses were performed in SAS statistical software (SAS package, S.A.S. Inst. 2007).

## Results

**Figure 1:**
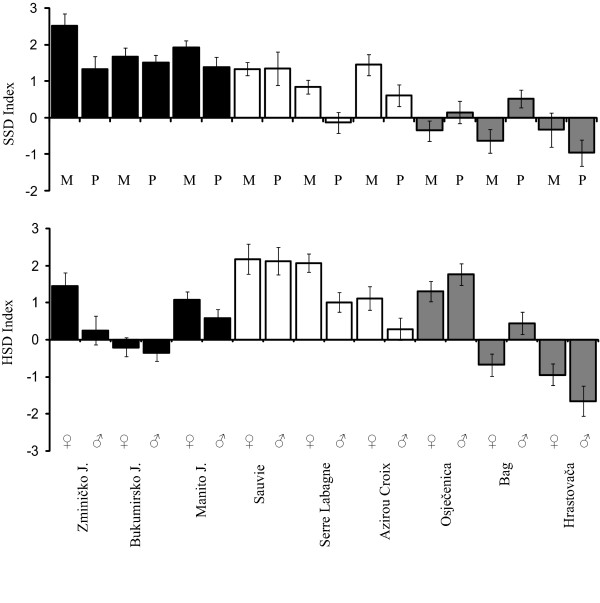
**Sexual size and heterochronic dimorphism indexes**. The sexual size dimorphism index (SSD I _PC1 _= female PC1 - male PC1) and the heterochronic size dimorphism index (HSD I _PC1 _= metamorph PC1 - paedomorph PC1). Values are means ± SE, derived from the distribution of 1000 bootstrap estimates. Full bars: the alpine newt *Mesotriton alpestris*; open bars: the palmate newt *Lissotriton helveticus*; grey bars: the smooth newt *L. vulgaris*, met = metamorphs, paed = paedomorphs, and J. = Jezero (lake). See Table 2 for the results of the statistical tests.

The body size of paedomorphs and metamorphs was characterised by a set of seven linear measurements. To explore size dimorphism between sexes and morphs we performed a principal components analysis (PCA). The first principal component (PC1) explained the largest proportion of overall variation (66%). All analysed morphometric traits, except head length (HL), had similar loadings on the first axis and evenly contribute to variation in body size. Therefore, the individual scores on PC1 were used to estimate the differences in overall body size. As our sample consists of sub-samples that largely differ in sample size (Table 1), we applied a modified bootstrap procedure to evaluate the precision of the calculated SSD indices and indices of differences in size between morphs (HSD indices). The means and standard errors of bootstrapped samples showed that population estimates of the original sample fall well within the range of estimates for bootstrapped samples (Table 2, Figure 1). Therefore, further analyses were performed on the original dataset. An analysis of variance indicates that population, morph, and sex have significant effects on body size (Table 3). Moreover, the significant morph × sex interaction indicates a divergence of sexual size dimorphism between morphs. A significant morph × population interaction was found in all three species as well, which indicates that the effects of morph type on SSD depend on the population source.

**Table 2:** The multivariate sexual size dimorphism Iindex SSD I_PC1 _and the heterochronic size dimorphism index HSD I_PC1 _(ANOVAs on PC1 scores)

	Metamorphs		Paedomorphs		Females		Males	
Species and population	SSD I_PC1_	*P*	SSD I_PC1_	*P*	HSD I_PC1_	*P*	HSD I_PC1_	*P*
**Alpine newt**								
Zminičko lake	2.515	**0.0001**	1.319	**0.0041**	1.485	**0.0008**	0.288	0.4969
Bukumirsko lake	1.664	**0.0001**	1.505	**0.0001**	-0.170	0.4229	-0.632	0.0752
Manito lake	1.922	**0.0001**	1.373	**0.0001**	1.145	**0.0001**	0.596	**0.0002**
								
**Palmate newt**								
Sauvie	1.334	**0.0001**	1.346	**0.0181**	2.168	**0.0001**	2.179	**0.0001**
Serre de la Labagne	0.842	**0.0120**	-0.137	0.6934	2.043	**0.0001**	1.064	**0.0015**
Azirou-croix	1.444	**0.0001**	0.608	0.0890	1.115	**0.0022**	0.279	0.3880
								
**Smooth newt**								
Velika Osječenica	-0.331	**0.0317**	-0.956	0.3658	1.316	**0.0001**	1.818	**0.0001**
Bag	-0.353	0.0681	0.149	**0.0017**	-0.673	**0.0046**	0.488	**0.0499**
Hrastovača	-0.638	0.4623	0.523	**0.0027**	-0.946	**0.0145**	-1.570	**0.0001**

**Table 3:** The effects of population, sex and morph and their interactions on body size (estimated as individual scores on PC1 scores; ANOVA).

Source		Alpine newt			Palmate newt			Smooth newt		
	df	SS	*F*	*P*	SS	*F*	*P*	SS	*F*	*P*
Pop	2	10.04	9.10	**0.0001**	15.04	8.49	**0.0003**	709.97	454.56	**0.0001**
Sex	1	183.87	333.09	**0.0001**	36.96	41.72	**<0.0001**	5.44	6.96	**0.0087**
Morph	1	15.77	28.56	**0.0001**	97.86	110.47	**<0.0001**	0.39	0.51	0.4776
Population × sex	2	0.96	0.87	0.4200	7.63	4.31	**0.0150**	5.10	3.27	**0.0393**
Population × morph	2	21.21	19.21	**0.0001**	16.50	9.31	**0.0001**	126.74	81.15	**0.0001**
Morph × sex	1	6.28	11.38	**0.0008**	4.07	4.59	**0.0335**	2.27	2.91	0.0889
Population × morph × sex	2	2.21	2.01	0.1361	2.15	1.21	0.2997	8.71	5.58	**0.0041**

Errors df: 355 (alpine newt), 168 (palmate newt), 373 (smooth newt).

### Sexual size dimorphism

There was a statistically significant female-biased SSD in the two morphs of all the alpine newt populations (Table 2, Figure 1). In the palmate newt, a significant female-biased SSD was recorded among metamorphosed individuals in the three analysed populations, but only in one population for the paedomorphs (Table 2, Figure 1). In the smooth newt, statistically significant male biased SSD in metamorphs was found only in one population, while paedomorphic males were significantly larger than paedomorphic females in two populations (Table 2, Figure 1).

### Size differences between morphs

Depending on the population source, paedomorphs and metamorphs differed in body size. In the alpine newt, this concerned both sexes in one population and females only in another, while in the third population no body size difference between sexes was found (Table 2, Figure 1). In the palmate newt, metamorphs were larger than paedomorphs, except for one population, in which males of both morphs attained approximately the same size (Table 2, Figure 1). In contrast, the smooth newt exhibited a diversified pattern. Metamorphs were larger than paedomorphs in both sexes in one population, smaller than paedomorphs in another, and in the third population, metamorphs were smaller than paedomorphs in females and larger than paedomorphs in males (Table 2; Figure 1).

### Effect of paedomorphosis on SSD

**Figure 2:**
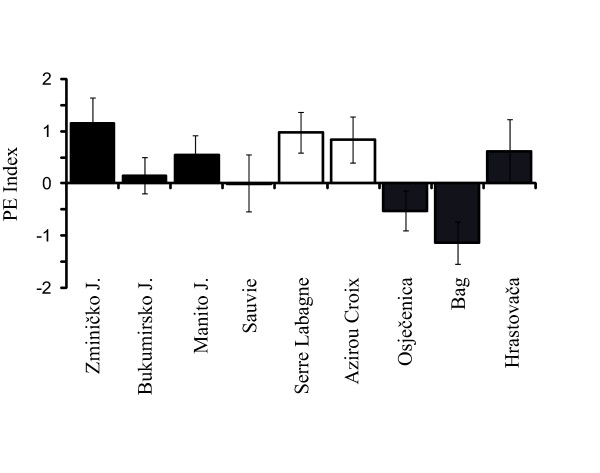
**Paedomorphosis index**. Paedomorphosis effects on SSD estimated as the paedomorphosis index (PE = SSD I _PC1 _metamorphs - SSD I _PC1_paedomorphs). Values are means ± SE derived from the distribution of 1000 bootstrap estimates. J. = Jezero (lake). See the legend of Figure 1 for complete details.

In the Alpine newt, the level of SSD in paedomorphs was about 50% lower than that of metamorphs in the population of Zminičko Jezero (Table 2, Fig 1). In this population, the bootstrap test against the null hypothesis of no differences in SSD between morphs (i.e., the paedomorphosis effect index) found that SSD Indices significantly differ (*P *< 0.05; Figure 2). The SSD in paedomorphs of the other populations was lower than for the metamorphs, although the differences were not significant. In the palmate newt, a significant reduction of SSD occurred at Serre de la Labagne (*P *< 0.05; Figure 2), but in the other two populations no statistically significant differences in SSD level between morphs were found. In the smooth newt, paedomorphosis was significantly associated with a change in the direction, but not the magnitude, of SSD (population of Bag, with a male-biased SSD in metamorphs, and a female-biased SSD in paedomorphs, *P *< 0.05; Figure 2). In the two other populations of smooth newt, the magnitude of SSD varied in paedomorphs relative to metamorphs, with increased SSD among paedomorphs in one population and decreased SSD in the other (Table 2, Figure 1). However, the observed changes between morphs in these two smooth newt populations were not statistically significant.

## Discussion and conclusion

Our data point out the importance of developmental processes in shaping SSD patterns in individuals that follow different life-history pathways. Paedomorphic and metamorphic European newts express contrasting patterns of SSD magnitude and direction depending on species and population. In the species with consistent female-biased SSD (i.e., the alpine newt) and in the species with slightly variable SSD (i.e., the palmate newt), paedomorphosis was associated with reduced SSD and similar sizes of females and males. However, in the species with the most varied SSD (i.e., the smooth newt), the effects of morph type on SSD are also more variable because paedomorphosis changes both the magnitude and the direction of SSD in a population-specific manner. These findings highlight the importance of studying alternative morphotypes in a wide range of animal groups, such as horned beetles [5], marine isopods [23], acarid mites [24], and fish [25], that exhibit intrasexual alternative developmental pathways associated with contrasting SSD patterns. In these polymorphic species, one male morph frequently exhibits less conspicuous sexually dimorphic characters than the other morph and is consequently more like females (for instance, small horns in horned beetles [5]). Numerous proximate and ultimate mechanisms that produce SSD are often not well understood because of their complex effects and interactions [[1] and references herein, [2,5,17,26-33]]. Here, we extend results of previous SSD studies in showing that intraspecific patterns of alternative ways of development need to be integrated in evolutionary models considering such species.

Variation of SSD according to alternative ontogenetic pathways (i.e., paedomorphosis vs. metamorphosis) can originate partly from the magnitude of the size differences between the sexes. In this regard, Griffith [7] showed that squamate species that display heterochronic patterns leading to a larger body size induced higher sexual size dimorphism. An additional factor that can affect the extent of SSD relies on the ontogenetic processes that can produce heterochronic morphs [9,10]. In tailed amphibian paedomorphosis, both progenesis and neoteny were identified [34]. Because progenesis corresponds to an acceleration of sexual maturation and neoteny to a reduction of somatic development, paedomorphs produced by the two processes follow different developmental pathways and are expected to differ greatly in body size at maturity. Progenetic newts can mature years before larvae that metamorphose into juveniles and mature on land whereas neotenic newts attain sexual maturity at the same age as their metamorphic counterparts [34,35]. Because of these contrasted ontogenies, progenetic, but usually not neotenic, paedomorphs were found to be smaller than metamorphs. To distinguish between heterochronic processes, ontogenetic data are necessary. However, from the size differences between metamorphs and paedomorphs in the present study, it is likely that the two processes (progenesis and neoteny) are acting on the studied populations. The only data related to the ontogenetic growth aspects of SSD in newts are from the group of European newts (crested newts). In these newts, newly metamorphosed juveniles do not exhibit SSD. However, juvenile females and males differ in growth rate, and SSD in these newts develops before sexual maturity [36]. Differences in growth rate appear in the period between the first and second hibernations. Therefore, we could expect that progenesis (sexual maturation at earlier age) will lead to reduced SSD level in paedomorphs [36]. For the populations that exhibit neoteny (at least for the one population of alpine newt in which paedomorphs and metamorphs do not differ in body size: Bukumirsko Jezero), no associations between SSD and intrasexual polymorphism were found. Such an effect of body size on morph variation was also highlighted in horned beetles, where small males are constrained to exhibit small horns and thus a smaller SSD pattern than large males [5].

Despite the trends for each species, large variation across populations was found within each species with respect to the effect of paedomorphosis on SSD. This finding is not very surprising because the growth and life-history of newts (age at maturity, paedomorphosis vs. metamorphosis) rely on environmental factors that will vary across populations, such as temperature [18], drying risk [37], and food availability [38,39].

Although further study is needed to elucidate the mechanisms that underlie the obtained patterns, our results offer two general lessons. First, the importance of studying alternative morphotypes, which are found in a wide range of animal groups, to understand the evolution of SDD. Second, understanding the link between body size variation and paedomorphosis is crucial to explain the ultimate advantages of alternative morphs.

## Authors' contributions

These authors contributed equally to this work. All authors read and approved the final manuscript.
